# Alcohol use in a military population deployed in combat areas: a cross sectional study

**DOI:** 10.1186/1747-597X-7-24

**Published:** 2012-06-13

**Authors:** Raveen Hanwella, Varuni Asanka de Silva, Nicholas E L W Jayasekera

**Affiliations:** 1 Department of Psychological Medicine, University of Colombo, Kynsey Road, Colombo, 08, Sri Lanka; 2 Department of Psychological Medicine, University of Colombo, Kynsey Road, Colombo, 08, Sri Lanka; 3 Director General Health Services, Sri Lanka Navy, Sri Lanka

**Keywords:** Sri Lanka, Alcohol, Military Personnel, War, Functional Impairment

## Abstract

**Background:**

Alcohol misuse is more prevalent among military populations. Association between PTSD and heavy drinking have been reported in many studies. Most of the studies on alcohol use among military personnel are from US and UK. Aim of this study is to describe alcohol consumption patterns among military personnel in Sri Lanka, a country where the alcohol consumption among the general population are very different to that in US and UK.

**Methods:**

Cross sectional study consisting of representative samples of Sri Lanka Navy Special Forces and regular forces deployed in combat areas continuously during a one year period was carried out. Data was collected using a self report questionnaire. Alcohol Use Disorder Identification Test (AUDIT) was used to assess alcohol consumption.

**Results:**

Sample consisted of 259 Special Forces and 412 regular navy personnel. The median AUDIT score was 2.0 (interquartile range 6.0). Prevalence of current drinking was 71.2 %. Of the current users 54.81 % were infrequent users (frequency ≤ once a month) while 37.87 % of users consumed 2–4 times a month. Prevalence of hazardous drinking (AUDIT ≥ 8) was 16.69 % and binge drinking 14.01 %. Five (0.75 %) had AUDIT total ≥20. There was no significant difference between Special Forces and regular forces in hazardous drinking or binge drinking. Total AUDIT score ≥16 were associated with difficulty performing work.

**Conclusions:**

High rates of hazardous drinking and binge drinking described among military personnel in US and UK were not seen among SLN personnel deployed in combat areas. This finding contrasts with previously reported association between combat exposure and hazardous alcohol use among military personnel. Alcohol use among military personnel may be significantly influenced by alcohol consumption patterns among the general population, access to alcohol and attitudes about alcohol use. Similar to findings from other countries, heavy alcohol use was associated with poorer psychological health and functional impairment.

## Background

Different studies in the United Kingdom (UK) and United States (US) armed forces show that alcohol misuse is more prevalent among military populations. A study of UK armed forces in service at the time of the Iraq War of 2003 reported that 67 % of men and 49 % of women had AUDIT scores >8 which indicate hazardous drinking [1]. These rates are higher than in the general population in that country. Among US Army soldiers deployed to Iraq and Afghanistan, in the post deployment period, 12.4 % of active component and 14.5 % of National Guard misused alcohol [2]. Following deployment there was also a change in the overall pattern of drinking with heavy drinking and binge drinking becoming more prevalent [3].The 2008 Department of Defence Health Behavior Survey conducted among US active duty personnel, reports that heavy alcohol use has increased significantly between 1998 (15 %) and 2008 (20 %) [4].

Patterns of alcohol consumption among the general population in Sri Lanka is different to that of US and UK. According to the Global Status Report on Alcohol and Health, the abstinence rate among males in Sri Lanka (83 %) is much higher than in the US (28.3 %) or UK (10.4 %) [5]. The rate in Sri Lanka is confirmed by an epidemiological survey which found the prevalence of abstinence to be 56.5 % among urban males and 75.2 % among rural males [6]. Among male drinkers per capita consumption of pure alcohol was less in Sri Lanka (9.32 litres) than in UK (21.58 litres) and US (19.98 liters) [5].

Factors which influence alcohol use in the military may be different to that among the general population. Among military personnel, there is evidence of association between combat stress and alcohol misuse [7-9]. In the US, Reserve and National Guard personnel and younger service members with combat exposures in Iraq and Afghanistan were at higher risk of alcohol problems [3]. In the UK military, binge drinking was more common among Army personnel, smokers and those who are younger and single [1].

Alcohol may be used as a maladaptive coping mechanism in stressful situations. In addition to combat stress, the drinking culture and alcohol policies in the military too influence alcohol consumption. Workplace culture in the military can be a risk factor for heavy alcohol consumption [10]. A culture that sanctions binge drinking and heavy drinking increases overall consumption. The reverse is also true. Bray et al. report that US military personnel deployed to the Persian Gulf reduced their use of alcohol, consistent with cultural prohibitions in the Persian Gulf region against alcohol use [11].

Heavy drinking among military personnel is associated with accidents, violence and self harm. It is also associated with impaired functioning and loss of productivity. Alcohol dependence and a high AUDIT score (20 or more) are associated with impairment such as physical and emotional problems interfering with social life, and reduction in work performance [12]. Other studies report association between drinking levels and loss in productivity [13].

The Sri Lanka Defence Forces was engaged in combat operations for 30 years. In 2006, the level of combat operations intensified as reflected in the casualty figures [14]. During the period 2006–2009, 190 officers and 5,700 other ranks of the Sri Lanka Army were killed and 27,000 injured [15]. In the Sri Lanka Navy (SLN), 485 personnel were killed and 245 permanently disabled [16].

Most of the studies on alcohol use among military personnel are from US and UK. There is little known about alcohol use among military personnel deployed in combat areas in other countries. Therefore we studied the alcohol consumption patterns among military personnel in Sri Lanka, a country where the patterns of alcohol consumption among the general population are very different to that in US and UK.

## Methods

### Study sample

A cross sectional study was carried out among representative samples of Sri Lanka Navy (SLN) Special Forces and regular forces deployed in combat areas to assess their mental health status [14]. Data collection commenced three months after combat operations ended. Only personnel who had served continuously in combat areas during the one year period prior to end of combat operations were included in the study. Both Special Forces and regular forces were selected using simple random sampling. The sample of SLN Special Forces was selected from the Special Boat Squadron which is the only Special Forces unit in the SLN. Since there are no females in the Special Forces, females were excluded from the regular forces group. The SLN central data base was used to identify all personnel who had served in combat areas continuously for at least one year. Participants were selected from this population using computer generated random numbers.

The participants thus identified were contacted at their workplace. They were briefed regarding the nature and objectives of the study by personnel from the medical corps. The briefings were carried out in small groups at the place of deployment to improve response rates, aid clarification and facilitate provision of support for those with mental health problems [14]. Participation was voluntary. The response rate was 93.8 %. The rate of missing values for individual items in the survey was about 10 %.

The sample size was calculated to detect an odds ratio of 2.0 for disorders with an estimated prevalence of 15 %, a power of 90 % and confidence of 95 % (two tailed). The required sample size was 240 in each group. The sample size was increased by 15 % to adjust for nonresponse. The comparison group (regular forces) was oversampled to include more combat troops. A total of 259 Special Forces and 412 regular navy personnel were recruited into the study [14].

### Outcome measures

The 28 page questionnaire used in the study “Health of UK military personnel deployed to the 2003 Iraq war” was used as the data collection instrument [17]. Permission was obtained from the authors for the use of the questionnaire. Alcohol Use Disorder Identification Test (AUDIT) developed by the World Health Organization as a simple method of screening for excessive drinking and to assist in brief assessment was used to assess alcohol use [18]. The AUDIT consists of 10 questions and each response has a score ranging from 0 to 4. The total AUDIT score reflects the person’s level of risk related to alcohol consumption. Hazardous alcohol use was defined as AUDIT total ≥8. High level of alcohol problems was defined as AUDIT total 16 ≥. Probable dependence was defined as AUDIT ≥20. Binge drinking was defined as consumption of 6 or more drinks on one occasion at least once a month.

Symptoms of common mental disorders were identified using the General Health Questionnaire 12 (GHQ-12) and cases were defined as those scoring 4 or more [19]. Post Traumatic Stress Disorder (PTSD) was diagnosed using the 17-item National Centre for PTSD checklist civilian version (PCL-C) and cases were defined as a score of 50 or more [20].

The questionnaire included five questions from the SF-36 that specifically assesses functional impairment. These included one item of physical or emotional problems interfering with normal social activities with family, friends, neighbours, or groups, and four items of problems with work or other regular activities as a result of physical health [16].

### Ethical approval

Ethical clearance was obtained from the Ethics Review Committee of the Faculty of Medicine, University of Colombo. Participation was voluntary and written informed consent was obtained from all participants. The questionnaire did not identify the participants by name.

### Statistical analyses

Analyses were done using SPSS version 13.0. Logistic regression analysis was used to calculate unadjusted and adjusted odds ratios (OR) with 95 % confidence limits (95 % CI). Model adequacy was tested using goodness of fit with the Hosmer–Lemeshow test.

## Results

### Study sample

The sample consisted of 259 Special Forces and 412 regular navy personnel. The mean age of the sample was 27.63 years (SD 5.02). Three hundred and twenty nine (49.03 %) were single, 333 (49.62 %) were married and 2 (0.30 %) were previously married. There were 33 (4.92 %) commissioned officers, 104 (15.50 %) non commissioned officers and 534 (79.58 %) other ranks. Two hundred and thirty six (35.17 %) were engaged in combat duty, 195 (29.06 %) served on board naval vessels and 237 (35.32 %) were engaged in noncombat duties which included medical, logistic, engineering, communication and administrative roles [13].

### Alcohol use

Table 1 shows the categorisation of the sample according to AUDIT scores. Prevalence of current drinking was 71.23 %. Of the current users 54.81 % were infrequent users (frequency once a month or less) while 37.87 % of users consumed 2–4 times a month. The median AUDIT score was 2.0 (interquartile range 6.0). Prevalence of hazardous drinking (AUDIT ≥ 8) was 16.69 %. Five (0.75 %) had AUDIT total ≥20. Binge drinking at least once a month was reported by 94 (14.01 %). Only 16 (2.38 %) reported binge drinking weekly or more often. Prevalence of hazardous drinking was 37.50 % (95 % CI 10.86-64.14) among the 16 personnel who fulfilled criteria for PTSD. The comparative rate among those without PTSD was 16.18 % (95 % CI 13.36-19.01].

**Table 1:** Socio demographic characteristics and AUDIT scores

	**AUDIT < 8 n(%)**	**AUDIT 8**–**15 n(%)**	**AUDIT 16**–**19 n(%)**	**AUDIT ≥20 n(%)**	**Binge drinking n(%)**
**Service type**					
Special Forces	214 (82.62)	33 (12.74)	11 (4.24)	1 (0.38)	39 (15.06)
Regular Forces	344 (83.49)	46 (11.16)	18 (4.36)	4 (0.97)	55 (13.35)
**Age (years)**					
≤ 30	415 (83.84)	54 (10.90)	23 (4.65)	3 (0.61)	75 (15.15)
>30	143 (81.25)	25 (14.20)	6 (3.41)	2 (1.14)	19 (10.80)
**Marital Status**					
Never married	274 (82.28)	37 (11.11)	19 (5.71)	3 (0.90)	53 (15.91)
Married/divorced	284 (84.02)	42 (12.43)	10 (2.96)	2 (0.59)	41 (12.13)
**Educational Status**					
Less than GCE O’Level	204 (83.61)	30 (12.30)	9 (3.69)	1 (0.41)	36 (14.75)
GCE O Level	243 (84.08)	34 (11.76)	10 (3.46)	2 (0.69)	39 (13.49)
GCE A Level or higher	111 (80.43)	15 (10.87)	10 (7.25)	2 (1.45)	19 (13.77)
**Rank Current**					
Commissioned Officer	28 (84.85)	3(9.09)	1(3.03)	1(3.03)	4 (12.12)
Non commissioned officer	88 (84.62)	12(11.54)	3(2.88)	1(0.96)	10 (9.61)
Other ranks	442 (82.77)	64(11.99)	25(4.68)	3(0.56)	80 (14.98)

Odds ratios were calculated for associations between the hazardous alcohol use and demographic variables using multivariable logistic regression analysis (Table 2). We adjusted for smoking status and GHQ caseness. There was no significant difference in hazardous drinking between Special Forces and regular forces. There was no significant difference in risk of binge drinking between Special Forces and regular forces. Unadjusted odds ratios show that being a current smoker and GHQ caseness were associated with hazardous drinking.

**Table 2:** Association between socio-demographic variables and hazardous drinking calculated using multivariable logistic regression analysis

	**AUDIT hazardous drinking AUDIT ≥8 number (%)**	**Unadjusted OR (95 % CI) 5**	***Adjusted OR (95 % CI)
Service type*		Wald 0.09	Wald 0.46
		p = 0.77	p = 0.83
Regular Forces	68 (16.50)	1.0	1.0
Special Forces	45 (17.37)	1.06(0.70-1.61)	1.05 (0.68-1.62)
Age (years)*		Wald 0.62	Wald 0.87
		p = 0.43	p = 0.35
≤ 30	80 (16.16)	1.0	1.0
>30	33 (18.75)	1.21 (0.77-1.87)	1.25 (0.79-1.98)
Marital Status*		Wald 0.36	Wald 0.08,
		p = 0.54	p = 0.78
Never married	59 (17.71)	1.0	1.0
Married/divorced	54 (15.97)	0.88 (0.59-1.32)	0.94 (0.62-1.43)
Educational Status**		Wald 0.94	Wald 0.42,
		p = 0.63	p = 0.81
<GCE O’Level	40 (16.39)	1.0	1.0
GCE O Level	46 (15.91)	0.97(0.61-1.53)	0.94 (0.58-1.52)
≥GCE A Level	27 (19.56)	1.24(0.72-2.13)	1.12 (0.64-1.97)
Rank (Current)**		Wald 0.28	Wald 0.22
		p = 0.87	p = 0.90
Commissioned Officer	5 (15.15)	0.87 (0.33-2.28)	0.89 (0.33-2.38)
Non-commissioned Officer	16 (15.38)	0.89 (0.50-1.58)	0.89 (0.49-1.60)
Other ranks	92(17.22)	1.0	1.0
Role within unit**		Wald 3.94	Wald 3.15
		p = 0.14	p = 0.21
Land combat	45 (19.07)	1.0	1.0
On board naval vessels	24 (12.30)	0.60 (0.35-1.02)	0.62 (0.36-1.08)
Others	43 (18.14)	0.94 (0.59-1.50)	0.93 (0.57-1.52)
Smoking status*		Wald p = 23.93	Wald 22.17,
		p < 0.001	p < 0.001
Non smoker	74 (13.43)	1.0	1.0
Current smoker	39 (32.50)	3.10 (1.97-4.89)	3.02 (1.91-4.78)
GHQ Case*		Wald 13.23	Wald 11.38
		p < 0.001	p < 0.001
GHQ case no	87 (14.69)	1.0	1.0
GHQ case yes	25 (31.64)	2.65 (1.57-4.48)	2.53 (1.48-4.34)

***Adjusted for smoking status and GHQ caseness.

*df = 1, ** df = 2.

### Functional impairment according to alcohol consumption

Unadjusted odds ratios obtained using logistic regression analysis indicate the association between different AUDIT domains and functional impairment (Table 3). Total AUDIT score ≥8 and binge drinking were not associated with functional impairment. Total AUDIT score ≥16 was significantly associated with limitations in type of work and difficulty performing work.

**Table 3:** Association between AUDIT scores and functional impairment

	**Total sample n (%)**	**AUDIT ≥ 8**	**AUDIT ≥ 16**	**Binge drinking**
		**n = 112**	**n = 34**	**n = 94**
		**OR, Wald, significance**	**OR, Wald, significance**	**OR, Wald, significance**
Health interfered with social life	157 (23.40)	OR1.54 (0.98-2.42) Wald 3.60, p = 0.06	OR1.85 (0.89-3.83) Wald 2.75, p = 0.10	OR 1.0 (0.60-1.67) Wald 0.0,p = 0.99
Cut down time on work/other activities	82 (12.22)	OR1.48 (0.84-2.61) Wald 1.84, p = 0.18	OR 1.25 (0.47-3.34) Wald 0.21,p = 0.65	OR 1.18 (0.63-2.23) Wald 0.26, p = 0.61
Accomplished less than would like	89 (13.26)	OR1.11 (0.62-1.99) Wald 0.12, p = 0.73	OR 0.87 (0.30-2.52) Wald 0.07, p = 0.79	OR 1.06 (0.56-2.10) Wald 0.03,p = 0.86
Limited in type of work	101 (15.05)	OR1.19 (0.69-2.05) Wald 0.38, p = 0.54	OR 2.47 (1.14-5.33) Wald 5.29,p = 0.02	OR 1.17 (0.65-2.10) Wald 0.28, p = 0.60
Difficulty performing work	165 (24.59)	OR1.50 (0.96-2.34) Wald 3.19, p = 0.07	OR 2.56 (1.27-5.17) Wald 6.92, p = 0.009	OR 1.13 (0.69-1.86) Wald0.23, p = 0.63

Test used -Logistic regression analysis.

df = 1.

## Discussion

Our study had several important findings. Prevalence of hazardous use and binge drinking was lower compared to US and UK armed services personnel [1,3,12,13,21,22]. There was no significant difference in risk of hazardous alcohol use or binge drinking between Special Forces and regular forces. AUDIT score ≥16 were significantly associated with functional impairment while hazardous use and binge drinking were not.

Although prevalence of alcohol consumption was higher among SLN personnel compared to the general population in Sri Lanka, majority of users in the SLN consumed alcohol less frequently than once a month [6,23]. Among SLN personnel the prevalence of hazardous use was 16.69 %. We do not have comparable data based on the AUDIT scale for the general population. The only data indicating harmful alcohol use among the general population is that 14.8 % of urban males consume more than 14 units of alcohol per week [6]. There is no data on prevalence of binge drinking among the general population in Sri Lanka.

Pattern of alcohol use among our study sample was very different to that of Western military personnel. Unlike in the UK armed Forces, alcohol misuse was not a significant problem in the SLN [12]. Hazardous alcohol use (AUDIT ≥8) was much less prevalent among SLN personnel (16.69 %) compared to US National Guard recently returned from duty in Afghanistan and Iraq (36 %) and UK armed services personnel in service in Iraq (67 %) [12,24]. Prevalence of binge drinking too was lower in our sample (26.1 %) compared to active duty US military personnel (43.2 %) and US Navy personnel (40.1 %) [22]. The frequency of binge drinking too was lower. Weekly binge drinking was reported among 46 % of UK armed forces personnel compared to 2.38 % in the SLN [12].

In our sample only factors associated with hazardous drinking were being a current smoker and GHQ caseness. Although other studies have reported that younger personnel are at higher risk of hazardous alcohol use, we found that hazardous alcohol use was higher in those aged > 30 years [1,12]. This may be because in the Sri Lanka Navy the lower ranks have less access to alcohol. Similar to findings from other studies a significant association was seen between current smoking and hazardous alcohol use [1,25]. A previous study among the general population in Sri Lanka showed that smokers are more likely to be drinkers and vice versa [23]. There is evidence that liability to dependence of alcohol, tobacco and cannabis may be genetically mediated. In addition common environmental factors also influence comorbidity. For example smoking is a behaviour indulged in situations where alcohol is consumed. Higher AUDIT scores are known to be associated with GHQ and PTSD positive cases and with depression [2,12,26]. We too found that hazardous use was associated with GHQ caseness. The number of personnel who fulfilled criteria for diagnosis of PTSD were only 16 therefore we could not establish and association between PTSD and hazardous drinking. However among the 16 personnel identified as having probable PTSD, prevalence of hazardous drinking was double that of personnel without PTSD.

Association between alcohol consumption and functional impairment was similar to findings from UK [12]. AUDIT score ≥ 8 or binge drinking were not associated with functional impairment. Lack of association between binge drinking and functional impairment may be due to the low frequency of binge drinking in our sample. Total AUDIT score ≥16 were significantly associated with difficulty performing work and limitation in the type of work performed.

Our findings suggest that alcohol consumption patterns among the general population may significantly influence alcohol use among military personnel. For example binge drinking which was much more prevalent among US and UK military samples is also prevalent among young people in the general populations of those countries [27-29].

Socio cultural factors and policies regarding alcohol use in the military are also known to influence alcohol consumption. Workplace culture can influence beliefs about acceptable drinking contexts as well as drinking behaviour such as number of drinks consumed [30]. A climate conducive to drinking in the military can be shaped by the attitudes and norms regarding alcohol use and abuse that are shared and understood by the personnel [31]. In the military where personnel live and work together for prolonged periods the ‘drinking culture’ among personnel may exert significant influence on behaviour [21]. The culture in the SLN may not sanction heavy drinking and binge drinking which may explain the lower prevalence of these behaviours.

Officially too the Sri Lanka Navy does not sanction heavy drinking. The amount of alcohol available in the camps is restricted by a bar credit limit for four categories of personnel based on seniority. Consumption above the approved credit limit leads to an inquiry and cancellation of bar credit for repeated offenders.

Limited availability of alcohol too can influence consumption. The sample was drawn from personnel deployed in combat areas. Many of these personnel could not travel out of their camps except when on home leave. This restricted their access to alcohol to what was available in the camps. Service needs too would have discouraged consumption. Almost all the Special Forces were involved in combat. Among the regular forces too many were engaged in land combat or patrol duty or served on board naval vessels [14]. Because these personnel were on active duty for many months at a time this would have discouraged alcohol use, as consumption while on duty is strictly prohibited.

The study has some limitations. We used a self report questionnaire rather than structured clinical interviews for collection of data*.* Although underreporting of alcohol use is a possibility there is evidence that self reports are valid and reliable [32]. Because of the cross sectional nature of the study we were unable to establish if heavy drinking leads to poorer psychological health or vice versa.

## Conclusions

High rates of hazardous drinking and binge drinking described among military personnel in US and UK were not seen among SLN personnel deployed in combat areas. This finding contrasts with previously reported association between combat exposure and hazardous alcohol use among military personnel. Alcohol use among military personnel may be significantly influenced by alcohol consumption patterns among the general population, access to alcohol and attitudes about alcohol use. Similar to findings from other countries, heavy alcohol use was associated with poorer psychological health and functional impairment.

Our findings have several implications. They question the assumption that hazardous drinking is common among military personnel deployed in combat areas. Studies must attempt to identify factors other than combat exposure which influence alcohol consumption. Military personnel need to maintain optimal physical and psychological health to cope with demands of their occupation. The finding that higher AUDIT scores are associated with functional impairment and psychological ill health highlights the need for prevention programs and screening, identifying high risk groups and plan interventions for such personnel in the military.

## Competing interests

RH and VAdeS are civilians who provide honorary clinical services to the Sri Lanka Navy. They do not receive any financial remuneration for their services. NELWJ is the Director General of Health services in the Sri Lanka Navy. The Sri Lanka Navy did not have any role in the design, conduct of the study or preparation of manuscript.

## Authors’ contributions

VAdeS and RH contributed to the design of the project, supervision of data collection, analysis of data and writing of the paper. NELWJ contributed to the design of the project and supervision of data collection. All authors have read the final draft and are in agreement with the content of the manuscript.

## References

[B1] Fear NT Iversen A Meltzer H Workman L Hull L Greenberg N Barker C Browne T Earnshaw M Horn O Jones M Murphy D Rona RJ Hotopf M Wessely S Patterns of drinking in the UK Armed Forces Addiction 2007 102 1749 59 10.1111/j.1360-0443.2007.01978.x 17935583

[B2] Thomas JL Wilk JE Riviere LA McGurk D Castro CA Hoge CW Prevalence of mental health problems and functional impairment among active component and National Guard soldiers 3 and 12 months following combat in Iraq Arch Gen Psychiatry 2010 67 6 614 623 10.1001/archgenpsychiatry.2010.54 20530011

[B3] Jacobson IG Ryan MA Hooper TI Smith TC Amoroso PJ Boyko EJ Gackstetter GD Wells TS Bell NS Alcohol use and alcohol-related problems before and after military combat deployment JAMA 2008 300 6 663 675 10.1001/jama.300.6.663 18698065 PMC2680184

[B4] Bray RM Pemberton MR Lane ME Hourani LL Mattiko MJ Babeu LA Substance use and mental health trends among U.S. military active duty personnel: key findings from the 2008 DoD Health Behavior Survey Mil Med 2010 175 6 390 399 20572470 10.7205/milmed-d-09-00132

[B5] World Health Organization Global status report on alcohol and health 2011 World Health Organization, Geneva

[B6] de Silva V Samarasinghe D Gunawardena N Alcohol and tobacco use among males in two districts in Sri Lanka Ceylon Med J 2009 54 4 119 124 20052853 10.4038/cmj.v54i4.1452

[B7] Milliken CS Auchterlonie JL Hoge CW Longitudinal assessment of mental health problems among active and reserve component soldiers returning from the Iraq war JAMA 2007 298 18 2141 2148 10.1001/jama.298.18.2141 18000197

[B8] Hoge CW Auchterlonie JL Milliken CS Mental health problems, use of mental health services, and attrition from military service after returning from deployment to Iraq or Afghanistan JAMA 2006 295 9) 1023 1032 16507803 10.1001/jama.295.9.1023

[B9] Hoge CW Castro CA Messer SC McGurk D Cotting DI Koffman RL Combat duty in Iraq and Afghanistan, mental health problems, and barriers to care N Engl J Med 2004 351 1 13 22 10.1056/NEJMoa040603 15229303

[B10] Ames G Cunradi C Alcohol use and preventing alcohol related problems among youth adults in the military Alcohol Res Health 2004 28 252 257

[B11] Bray RM KL Luckey JW Wheeless SC Iannacchione VG 1992 Department of Defense Survey of Health Related Behaviors Among Military Personnel Research Triangle Inst (RTI): Research Triangle Park NC 1992 1992 1992

[B12] Rona RJ Jones M Fear NT Hull L Hotopf M Wessely S Alcohol misuse and functional impairment in the UK Armed Forces: a population-based study Drug Alcohol Depend 2010 108 1–2 37 42 20047802 10.1016/j.drugalcdep.2009.11.014

[B13] Mattiko MJ Olmsted KL Brown JM Bray RM Alcohol use and negative consequences among active duty military personnel Addict Behav 2011 36 6 608 614 10.1016/j.addbeh.2011.01.023 21376475

[B14] Hanwella R de Silva V Mental health of special forces personnel deployed in battle Soc Psychiatry Psychiatr Epidemiol 2012 47 8 1343 51 10.1007/s00127-011-0442-0 22038567

[B15] Ministry of Defence Wanni war heroes given military honours & their service appreciated[http://www.defence.lk/new.asp?fname=20090529_05]

[B16] Sri Lanka Navy Fallen comrades 2001–2005 [http://www.navy.lk/index.php?id=66]

[B17] Hotopf M Hull L Fear NT Browne T Horn O Iversen A Jones M Murphy D Bland D Earnshaw M Greenberg N Hughes JH Tate AR Dandeker C Rona R Wessely S The health of UK military personnel who deployed to the 2003 Iraq war: a cohort study Lancet 2006 367 1731 41 10.1016/S0140-6736(06)68662-5 16731268

[B18] World Health Organization Alcohol use disorder identification test 2001 World Health Organization, Geneva

[B19] Goldberg D WP: A users guide to the general health questionnaire 1988 NFER-Nelson, Windsor UK

[B20] Blanchard JAJ EB Buckley TC Forneris CA Psychometric properties of the PTSD checklist (PCL) Behav Res Ther 1996 34 669 673 10.1016/0005-7967(96)00033-2 8870294

[B21] Browne T Iversen A Hull L Workman L Barker C Horn O Jones M Murphy D Greenberg N Rona R Hotopf M Wessely S Fear NT How do experiences in Iraq affect alcohol use among male UK armed forces personnel? Occup Environ Med 2008 65 628 33 10.1136/oem.2007.036830 18178589

[B22] Stahre MA Brewer RD Fonseca VP Naimi TS Binge drinking among U.S. active-duty military personnel Am J Prev Med 2009 36 3 208 217 10.1016/j.amepre.2008.10.017 19215846

[B23] de Silva V Samarasinghe D Hanwella R Association between concurrent alcohol and tobacco use and poverty Drug Alcohol Rev 2011 30 1 69 73 10.1111/j.1465-3362.2010.00202.x 21219500

[B24] Burnett-Zeigler I Ilgen M Valenstein M Zivin K Gorman L Blow A Duffy S Chermack S Prevalence and correlates of alcohol misuse among returning Afghanistan and Iraq veterans Addict Behav 2011 36 8 801 806 10.1016/j.addbeh.2010.12.032 21482030

[B25] Iversen A Waterdrinker A Fear N Greenberg N Barker C Hotopf M Hull L Wessely S Factors associated with heavy alcohol consumption in the U.K. armed forces: data from a health survey of Gulf, Bosnia, and era veterans Mil Med 2007 172 9 956 961 17937359 10.7205/milmed.172.9.956

[B26] Jakupcak M Tull MT McDermott MJ Kaysen D Hunt S Simpson T PTSD symptom clusters in relationship to alcohol misuse among Iraq and Afghanistan war veterans seeking post-deployment VA health care Addict Behav 2010 35 9 840 843 10.1016/j.addbeh.2010.03.023 20471180

[B27] Courtney KE Polich J Binge drinking in young adults: Data, definitions, and determinants Psychol Bull 2009 135 1 142 156 19210057 10.1037/a0014414 PMC2748736

[B28] Wechsler H Lee JE Kuo M Seibring M Nelson TF Lee H Trends in college binge drinking during a period of increased prevention efforts. Findings from 4 Harvard School of Public Health College Alcohol Study surveys: 1993–2001 J Am Coll Health 2002 50 5 203 217 10.1080/07448480209595713 11990979

[B29] MacArthur GJ Smith MC Melotti R Heron J Macleod J Hickman M Kipping RR Campbell R Lewis G Patterns of alcohol use and multiple risk behaviour by gender during early and late adolescence: the ALSPAC cohort J Public Health (Oxf) 2012 34 Suppl 1 i20 30 10.1093/pubmed/fds006 22363027 PMC3284864

[B30] Ames GM Janes C Cultural approach to conceptualizing alcohol and the workplace Alcohol Health Res World 1992 16 2 112 119

[B31] Poehlman JA Schwerin MJ Pemberton MR Isenberg K Lane ME Aspinwall K Socio-cultural factors that foster use and abuse of alcohol among a sample of enlisted personnel at four Navy and Marine Corps installations Mil Med 2011 176 4 397 401 21539161 10.7205/milmed-d-10-00240

[B32] Williams GD Aitken SS Malin H Reliability of self-reported alcohol consumption in a general population survey J Stud Alcohol 1985 46 3 223 227 4010299 10.15288/jsa.1985.46.223

